# Resolution of plaque-type psoriasis: what is left behind (and reinitiates the disease)

**DOI:** 10.1007/s00281-019-00766-z

**Published:** 2019-10-31

**Authors:** Theresa Benezeder, Peter Wolf

**Affiliations:** 1 Department of Dermatology, Medical University of Graz, Auenbruggerplatz 8, 8036 Graz, Austria; 2 Center for Medical Research, Medical University of Graz, Graz, Austria

**Keywords:** IL-17, IL-23, Phototherapy, Tissue-resident memory T cells (TRMs), Molecular scar, Recurrence, Psoriasis

## Abstract

Psoriasis is a chronic inflammatory skin disease that involves numerous types of immune cells and cytokines resulting in an inflammatory feedback loop and hyperproliferation of the epidermis. A more detailed understanding of the underlying pathophysiology has revolutionized anti-psoriatic treatment and led to the development of various new drugs targeting key inflammatory cytokines such as IL-17A and IL-23. Successfully treated psoriatic lesions often resolve completely, leaving nothing visible to the naked eye. However, such lesions tend to recur within months at the exact same body sites. What is left behind at the cellular and molecular levels that potentially reinitiates psoriasis? Here, we elucidate the cellular and molecular “scar” and its imprints left after clinical resolution of psoriasis treated with anti-TNFα, anti-IL-17, or anti-IL-23 antibodies or phototherapy. Hidden cytokine stores and remaining tissue-resident memory T cells (TRMs) might hold the clue for disease recurrence.

## Background

Psoriasis is a chronic skin disease that results from a multifaceted interaction of immunological, environmental, and genetic factors [1, 2]. According to a recent systematic review, the global prevalence of psoriasis ranges from 0.09 to 5.1% [3]. The most common form of psoriasis, occurring in 85-90% of all patients, is plaque-type psoriasis characterized by well-demarcated, scaly and erythematous, infiltrated plaques [1, 4]. On the microscopic level, keratinocytes proliferate rapidly at high turnover rates, which leads to incomplete terminal differentiation. Abnormal differentiation causes thickening of the epidermis (acanthosis), retention of keratinocyte nuclei in the stratum corneum (parakeratosis), and loss of the granular layer. Neutrophils accumulate in the epidermis to form Munro’s microabscesses. Psoriatic lesions are highly vascular and also densely infiltrated by T cells and dendritic cells (DC) [2, 5, 6] which are key players in its pathophysiology.

## Pathophysiology of psoriasis

**Fig. 1:**
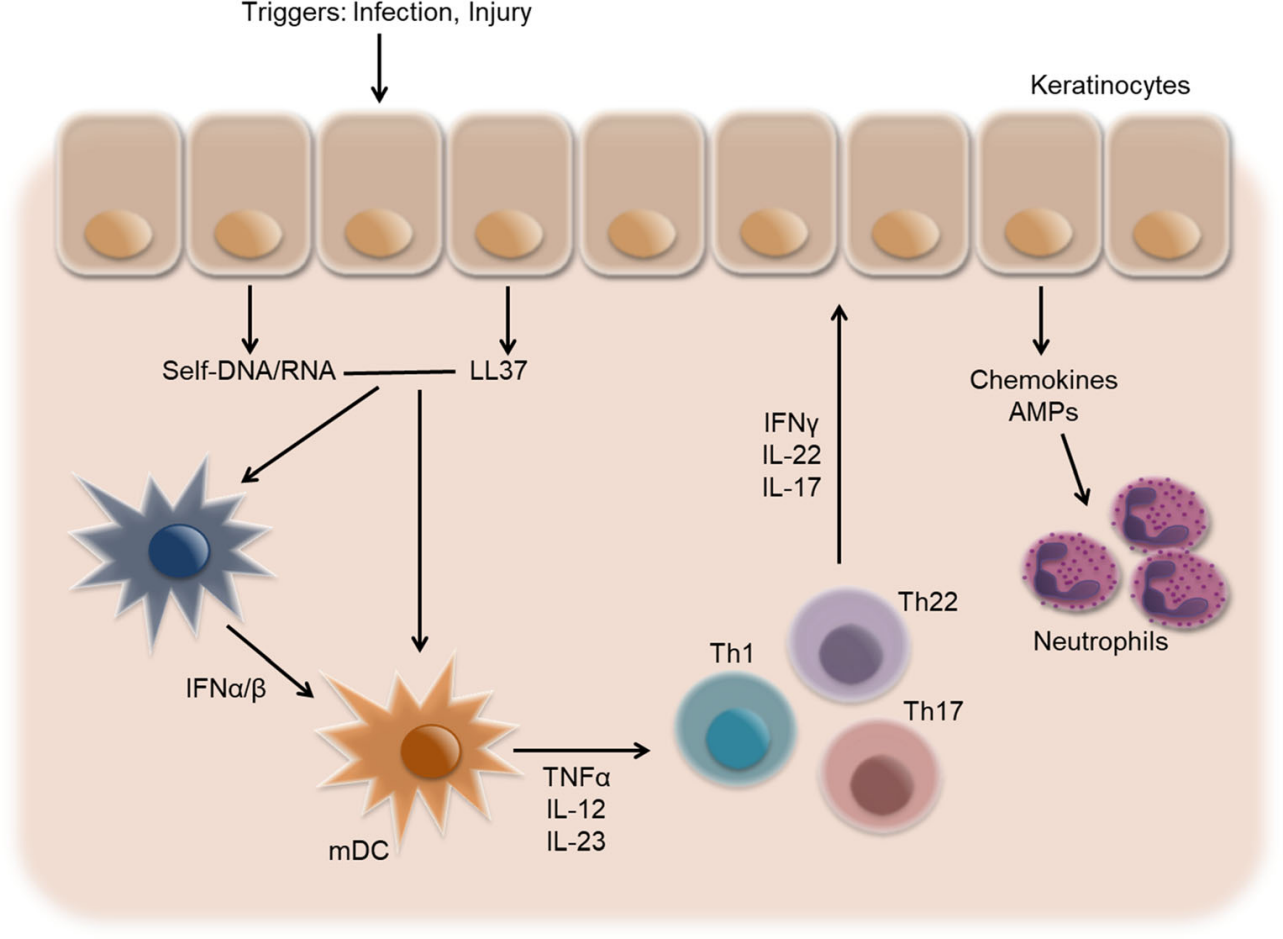
Initiation phase of psoriasis. Various triggers can cause activation of keratinocytes and the release of nucleic acids and antimicrobial peptides (e.g., LL-37), which form complexes and activate plasmacytoid dendritic cells (pDCs) and myeloid dendritic cells (mDCs). DCs promote differentiation of T cells into Th1, Th22, and Th17 subsets. Cytokines produced by these T cells such as IFNγ, IL-17, and IL-22 act on keratinocytes and cause hyperproliferation. Keratinocytes release AMPs and chemokines and attract neutrophils and other leukocytes

Various external factors like trauma and injury, infection, or medication can stress or damage keratinocytes [4]. Stressed or dying cells release nucleic acids along with the antimicrobial peptide (AMP) LL-37. LL-37 causes the loss of tolerance to self-nucleic acids and forms complexes with self-DNA/-RNA. These complexes can activate plasmacytoid dendritic cells (pDCs) via toll-like receptor 7 (TLR7) [7]. pDCs mainly produce type 1 interferons (IFN-α, IFN-β), which then activate myeloid dendritic cells (mDCs) to produce key psoriatic inflammatory cytokines IL-12 and IL-23 [8]. mDCs can also be directly activated by nucleic acid-LL37 complexes via TLR8 [9] and then migrate to lymph nodes where they promote the differentiation and activation of T cells via TNFα, IL-12, and IL-23. Activated T cells enter the circulation and move to inflamed skin via adhesion molecules on the endothelial cells of blood vessels. At the site of inflammation, different subsets of T cells release their effector molecules IFN-γ, IL-17, and IL-22. These cytokines, in concert with the pro-inflammatory signals such as IL-23 produced by mDCs, act on keratinocytes. Keratinocytes produce AMPs, cytokines, and chemokines to attract other immune cells like neutrophils and macrophages (Fig. 1) [2, 5, 10].

**Fig. 2:**
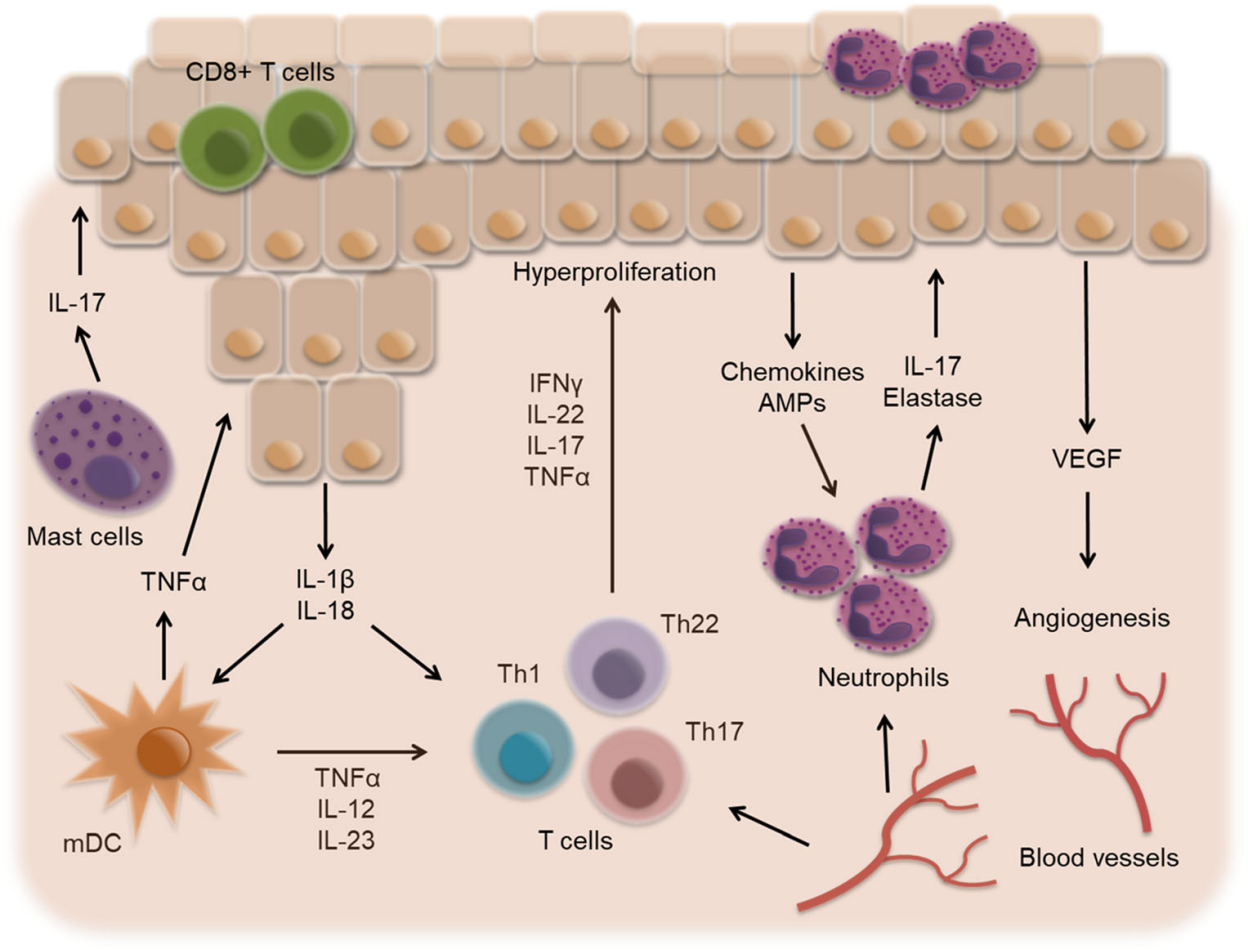
Chronic psoriatic lesion. In psoriasis, mast cells, neutrophils, myeloid dendritic cells (mDCs), and T cells produce pro-inflammatory cytokines. Proliferating keratinocytes release IL-1β, IL-18, chemoattractants, and VEGF resulting in accumulation of neutrophils in the epidermis and increased angiogenesis

In chronic psoriatic lesions, mDCs produce IL-12 and IL-23, which in turn constantly stimulate T cell subsets Th1, Th22, and Th17 to release IFNγ, IL-22, and IL-17. CD8+ T cells are also present in psoriasis, produce the same range of cytokines as CD4+ cells, and reside predominantly within the epidermis [5, 10].Over the last decade, it has been shown that the main source of IL-17 in psoriasis is not T cells, but rather innate cells like neutrophils and mast cells [11–14]. Neutrophils, for example, are a rich source of IL-17, AMPs, and elastase, all of which help keratinocytes hyperproliferate. Recent studies also implicate innate lymphoid cells (ILCs) as an additional source of IL-17 in psoriasis [15, 16]. IL-17 acts directly on keratinocytes, the main IL-17 receptor (IL-17R)-expressing cell type, but has only limited effects on their gene expression [17]. Synergism of IL-17 and TNFα is key to the full-blown effect on mRNA expression of pro-psoriatic genes (such as *DEFB4*, *S100A7*, *IL19*, *IL17C*, *CXCL8*, *CCL20*, *LCN2*) in keratinocytes [18]. In psoriasis, a combination of the inflammatory cytokines IL-17, TNFα, IL-22, and IFNγ drives keratinocyte hyperproliferation and cytokine and chemokine release [5]. Keratinocyte-produced IL-1β and IL-18 act on DCs and T cells, and chemokines such as CXCL-5 and CXCL-8, as well as AMPs, constantly attract neutrophils [4]. Keratinocytes also produce vascular endothelial growth factor (VEGF), which recruits and favors proliferation of endothelial cells, thereby promoting angiogenesis and creating highly vascular psoriatic plaques (Fig. 2) [10]. Together, structural abnormalities of the epidermis, inflammatory cellular skin infiltration, and increased dermal angiogenesis result in full-blown chronic psoriasis.

## Treatments leading to long-lasting clearance of psoriasis

A more detailed understanding of the pathophysiology of psoriasis has revolutionized anti-psoriatic treatment and led to the successful development of various new drugs targeting key inflammatory cytokines (Table 1).

**Table 1:** Currently approved biologics for the treatment of psoriasis and their PASI75 response rates

Target	Name	Type	PASI75% (*n*)	Timepoint	Approval
TNFα	Adalimumab [19]	Monoclonal antibody	71% (578 of 814)	16 weeks	2008
	Etanercept [20, 21]	Soluble TNFα-receptor	47% (147 of 311)	12 weeks	2004
	Infliximab [22]	Monoclonal antibody	80% (242 of 301)	10 weeks	2006
	Certolizumab Pegol [23]	PEGylated Fab′ fragment	83% (48 of 58)	12 weeks	2018
IL-12/IL-23p40	Ustekinumab [24]	Monoclonal antibody	76% (311 of 411)	12 weeks	2009
IL-23p19	Guselkumab [25]	Monoclonal antibody	91% (300 of 329)	16 weeks	2019
	Tildrakizumab [26]	Monoclonal antibody	A) 62% (192 of 308) B) 79% (236 of 298)	12 weeks (A) 28 weeks (B)	2018
	Risankizumab [27]	Monoclonal antibody	93% (77 of 83)	12 weeks	2019
IL-17A	Ixekizumab [28]	Monoclonal antibody	A: 98.6% (72 of 73) B: 100% (72)	12 weeks (A) 24 weeks (B)	2016
	Secukinumab [29]	Monoclonal antibody	82% (200 of 245), 77% (249 of 323)	12 weeks	2015
IL-17RA	Brodalumab [30]	Monoclonal antibody	83% (185 of 222)	12 weeks	2017

Nowadays, targeted therapy, in particular with antibodies against IL-17 or IL-23, permits complete clinical clearance of skin lesions in a high percentage of cases, at rates similar to those observed after PUVA treatment [31–35] but with an apparently more favorable risk profile. Severity of psoriasis is assessed with tools such as the psoriasis area and severity index (PASI), which scores both involved body area and clinical appearance of psoriatic lesions. In treated patients, complete clearance is observed in approximately 30 to 50% of patients and reduction in severity of symptoms by 90% (PASI90) in approximately 50 to 70% of patients [25, 27–29]. However, if treatment is stopped, skin lesions eventually reappear within months. Conspicuously, patients treated with psoralen plus UVA (PUVA) photochemotherapy or anti-IL-23 antibodies have the longest disease-free periods. In fact, median time to recurrence was 8 months for PUVA treatment in the study by Yones et al. [32], compared to similar sustained remission times after stopping anti-IL-17 or anti-IL-23 antibody treatment. For instance, after stopping secukinumab, the median time to relapse was 20 to 24 weeks [36]; after stopping ixekizumab, 20 weeks [37]; after stopping tildrakizumab, 20 weeks (as measured by PASI75) [26]; and after stopping guselkumab, approximately 15 weeks (as measured by PASI90) [38].

As early as 1994, Vallat et al. [39] provided a possible explanation for the longer remissions obtained with phototherapy than with other treatments such as then state-of-the-art cyclosporine. They showed that bath PUVA strongly suppressed both immunological and epidermal activation in psoriasis. Keratinocyte proteins such as keratin 16, filaggrin, and involucrin that were abnormally expressed in active psoriasis were more normally expressed after PUVA treatment. PUVA strongly suppressed epidermal and dermal CD4+ and CD8+ T cell infiltration of the skin, with virtual elimination of IL-2 receptor bearing activated T cells in some patients [39]. The authors proposed that these changes could be the cellular basis for the more sustained disease remission seen after PUVA treatment versus that seen after simple immune suppression by cyclosporine. The therapeutic mechanisms of action of phototherapy in psoriasis have been recently reviewed, emphasizing phototherapy’s dual action on both players in the disease (i.e., keratinocytes and skin-infiltrating immune cells) [40–42].

Phototherapy induces apoptosis and immunosuppression. However, it is not clear whether these two effects act simultaneously or independently [43]. In psoriasis, PUVA [44, 45] or UVB [46–48] may act by upregulating anti-inflammatory cytokines (IL-4 and IL-10) and downregulating pro-inflammatory cytokines (IL-8, IL-17, IL-22, IL-23, TNFα, and IFNγ). In addition, UV therapy can induce regulatory T cells (Tregs) [49, 50] and Langerhans cell emigration [51, 52]. However, it has also been shown that phototherapy leads to direct apoptosis of keratinocytes. Indeed, a study using a computational model of psoriatic epidermis combined with in vivo and in vitro experimentation revealed that apoptosis occurs in both stem and transit-amplifying cells and seems to be sufficient to explain UVB-induced plaque resolution [53].

The dual action of phototherapy (and of PUVA in particular) on keratinocytes and the immune system may be responsible for the sustained resolution of psoriatic disease after cessation of treatment. Having said that, the effect of phototherapy in psoriasis is limited to the site of exposure [31, 54], which suggests a crucial direct effect on keratinocytes (i.e., apoptosis) beyond the local and systemic effects on the immune system. We speculate that a dual effect of anti-IL-17 or anti-IL-23 antibody treatment on keratinocytes and immunocytes may also be responsible for the long-lasting, sustained response seen after treatment cessation. In contrast, other anti-psoriatic treatments such as topical steroids, vitamin D_3_ analogs, or anthralin [55–57], systemic traditional treatments such as cyclosporine or methotrexate, and even first-generation biologics (i.e., TNF antagonists) are usually associated with rather quick relapses within several weeks after treatment is stopped.

## Resolution of psoriasis: remaining molecular imprints

Nowadays, psoriasis is considered as systemic disease often associated with comorbidities such as metabolic syndrome, hypertension, elevated blood lipid levels, and cardiovascular disease [58]. Intriguingly, psoriatic plaques appear in most patients only at certain body locations, in particular at predilection sites such as the extensor surfaces of the extremities (knee and elbows) and/or other mechanically stressed locations such as the sacral region. Even more intriguingly, despite a general genetic predisposition, more than 35 psoriatic susceptibility genes are known so far [59], certain patients develop life-long psoriasis only on fixed, circumscribed, and sometimes solitary body sites. Psoriatic lesions can resolve entirely after treatment and always without scarring. However, when treatment is stopped, lesions recur most often at the exact same body sites. This raises an important question: what kind of residual “molecular scars” are left behind on a clinically cleared psoriatic lesion that eventually cause its reappearance. What those molecular scars look like after treatment with topical and traditional systemic agents is obscured by insufficient data, but what they look like after treatment with anti-TNFα, anti-IL-17, and anti-IL-23 antibodies is clearer thanks to recent studies of skin biopsy samples.

### Anti-TNFα treatment

As early as 2007, when the crucial role of Th17 cells in psoriasis was not fully appreciated, Zaba and colleagues studied the effects of the soluble TNFα receptor fusion protein etanercept in a clinical trial enrolling 20 patients at several time points of biopsy sampling during 12 weeks of treatment [60]. Responders and non-responders were grouped with regard to remaining epidermal thickness and normalization of keratinocyte hyperproliferation as measured by Ki67 and K16 protein and *KRT16* gene expression. Fast normalization of keratinocyte hyperproliferation along with reduced expression of Th17-related genes (*IL17*, *CCL20*, *DEFB4*) was observed after only 1 week of TNFα inhibition. In addition, inflammatory products of DCs (*INOS*, *TNF*, *IL20*, *IL23p40*) were reduced early in treated psoriatic plaques. Going along with a significantly reduced PASI score, there were diminished numbers of dendritic cells and T cells and macrophages as well as a repressed *IL22* mRNA expression in psoriatic plaques after 2 weeks of etanercept treatment. Interestingly enough, Zaba et al. found a rather delayed response of Th1-related genes (*IFNG*, *LTA1*). They therefore proposed a model mechanism of TNFα inhibition in psoriasis where inflammatory DCs are quickly inhibited, in turn repressing Th17 response and keratinocyte hyperproliferation. They also argued that a delayed effect on Th1 response is still needed to completely clear psoriasis [60]. In 2009, the same group of investigators published a comprehensive microarray study, extending their previous findings. Intriguingly, immediate response genes of TNFα inhibition were linked to myeloid cells (DCs, Langerhans cells), but not T cells. Resident DC genes were upregulated early during treatment (myeloid DC genes *CD1C* and *CD1E* and Langerhans cell genes *CD207* and *CD1A*), with CD1c and CD207 cell counts supporting these results by being low at baseline, increasing at week 2, and then decreasing at week 12 to baseline levels. Furthermore, they saw a rapid decrease of TNF-response genes *IL1B* and *CXCL8* in both responders and non-responders. At the late time point, TNFα and IFNγ pathway genes were downregulated in both responders and non-responders, but IL-17 pathway genes were only downregulated in responders. This led Zaba et al. to conclude that suppression of Th17 response genes is necessary for resolution of psoriatic disease [61].

In 2011, Suárez-Fariñas and colleagues [62] were the first to define the term “residual disease genomic profile” in psoriasis. They analyzed biopsy samples from clinical and histological responders to etanercept. Histologically, sampled skin was classified as “normal”, as epidermal thickness had improved by nearly 100% and CD3+ T cells, DCs and macrophage cell counts had returned to non-lesional levels. The exception was CD8+ T cell infiltration of the skin, which was only partially reduced (by 64%) after etanercept treatment. For transcriptional analyses, the expression of known psoriasis-associated genes in pretreatment lesions was compared with those in resolved lesions. Not surprisingly, the expression of many inflammatory and keratinocyte-related genes had improved by more than 90%. Although upregulation of T cell genes was reduced by 88%, that of important inflammatory genes like *IL22*, *IL17*, *IL12p35*, and *IFNG* was reduced to a lesser extent (by approximately 65%). A “residual disease genomic profile” was defined as psoriasis-associated genes improving less than 75%. This profile was composed of about 250 transcripts and was divided into two functional groups, inflammation-associated genes and structural genes (e.g., genes related to lymphatic endothelial cells). These findings suggested that inflammation does not completely resolve even when psoriatic lesions are clinically healed and that some residual CD8+ T cells remain in the skin. In addition, some structural abnormalities of the skin were not fully reversed.

In 2014, Johnston et al. investigated very early changes in lesional skin during TNF inhibition by etanercept [63]. In their study, 20 etanercept responders were studied and gene expression was analyzed using qPCR and microarray at baseline, after 1 day, 3 days, 7 days, and 2 weeks of TNF inhibition. There were no changes in mRNA expression of *IL17A*, *IL22*, and *IFNG* in the first week of therapy. In line with the findings of Zaba et al. [61], other IL-17 pathway-related genes were suppressed by etanercept. Interestingly, Johnston and colleagues were able to show downregulation of IL-17 receptor C (*IL17RC*) after only 1 day of TNFα inhibition. Next, they compared microarray results in their etanercept-treated patients with those in ixekizumab (anti-IL-17A)-treated patients and found an extensive overlap in downregulated genes. When normal human keratinocytes were treated with TNFα in vitro, mRNA and protein expression of *IL17RC* increased. By inhibiting *IL17RC* using short hairpin RNA (shRNA), effects of TNFα were suppressed, indicating that etanercept acted by blocking IL-17A signaling. However, etanercept obviously does not directly target Th17 cytokines because *IL17A*, *IL22*, and activated *STAT3* expression levels were still high in treated lesions and were downregulated only much later. Early effects of TNFα inhibition might include suppression of IL-17RC in keratinocytes, in turn leading to decreased IL-17A sensitivity in the tissue and thereby halting the inflammatory feedback loop [63]. Similar results were found by investigating the effects of the TNFα antibody adalimumab. Genes associated with keratinocyte hyperproliferation were normalized, and mRNA expression of Th17-associated cytokines was downregulated [64, 65]. Moreover, Bose et al. [66] reported from a study of the effects of etanercept, adalimumab, and infliximab on psoriatic lesions that the anti-TNF-modulated genes most closely associated with clinical improvement were those encoding CCR-7 and its ligand, CCL-19, as well as genes involved in dendritic cell maturation, T cell activation, and VEGF expression.

### Anti-IL-17 treatment

Currently, two IL-17A antagonists are available for the treatment of psoriasis: ixekizumab (approved in 2016) and secukinumab (approved in 2015). In 2012, when the role of T cell subsets in psoriasis was not yet fully defined, Krueger and colleagues hypothesized that Th17 cells might be crucial and studied effects of IL-17 inhibition using the anti-IL-17A antibody ixekizumab. As they demonstrated by immunohistochemistry, ixekizumab not only decreased keratinocyte hyperproliferation (K16, Ki-67) and skin infiltration by T cells (CD3) and dendritic cells (CD11c), but also suppressed release of keratinocyte-produced AMPs (LL-37, S100A7, S100A8, and BD2). They next investigated early changes in gene expression in the skin during IL-17 inhibition using qPCR. After 2 weeks, expression of *KRT16*, *IL17A*, *IL17F*, *IL22, IL23p19*, and *LCN* was decreased, and microarray analysis showed that 765 genes were already differentially expressed compared to baseline. Among them were *IL19* (inducer of epidermal hyperplasia); *CXCL8* and *CXCL1* (IL-17-induced neutrophil chemokines); *CCL20* (chemokine for Th7 cells and dendritic cells); *GZMB* (granzyme B; effector molecule of cytotoxic T cells); and *LCN* (IL-17-induced AMP). Next, transcriptional data on skin samples from ixekizumab-treated patients were compared to etanercept’s. Of 1200 psoriasis genes, about 600 were normalized at 2 weeks by ixekizumab, but only about 100 were normalized by etanercept, translating into an improvement of 70% with IL-17 inhibition versus 35% with TNF inhibition. Interestingly, IL-17 pathway-associated molecules such as *CXCL1*, *CXCL8*, *DEFB4*, and *LCN* correlated with improvement of psoriatic epidermis at week 2, but *IL17A* and *IL17F* did not. Furthermore, epidermal improvement was not linked to suppression of Th1- and Th22-associated genes. This led Krueger et al. to conclude that early improvement during ixekizumab therapy is linked to suppressing IL-17’s effect on keratinocytes and not to changes in T cell infiltration of the skin or cytokines produced by T cells [67].

In the last few years, evidence has accumulated that T cells are not the only cell type producing IL-17 in psoriasis [11–13][14]. To further investigate the various sources of IL-17, Reich et al. [68] analyzed effects of IL-17 inhibition in secukinumab-treated patients after 2 and 12 weeks of therapy. After 2 weeks, neutrophil counts in the skin and markers for epidermal hyperproliferation were reduced, accompanied by a reduction of expression of IL-17-induced neutrophil chemoattractants (*CXCL1*, *CXCL8*), which are produced by keratinocytes. A significant decrease in mRNA expression was also observed for *IL17A* and *IL17F*, but not for *TNF*. Interestingly, T cell and dendritic cell infiltration of the skin decreased more slowly. Immunostaining revealed that neutrophils were the predominant IL-17 source, followed by T cells and mast cells. However, mast cell numbers in the skin remained unchanged during and after secukinumab treatment. In comparison, the clinical result in a low-dose group in the same study was worse at 12 weeks and, strikingly, neutrophil counts and neutrophil chemoattractants were increased again. In general, patients with detectable neutrophil infiltration at 12 weeks had a shorter time to relapse than those who did not. These results led to the conclusion that neutrophils are an important source of IL-17 in psoriasis and that secukinumab suppresses crosstalk between keratinocytes and neutrophils. In this crosstalk, IL-17 produced and/or stored by neutrophils, mast cells, and T cells stimulates epidermal cells to produce neutrophil chemoattractants, leading to increased neutrophil counts in psoriasis. Inhibition of this crosstalk could be an early mechanism of secukinumab, while full clinical effects are linked to a decrease in T cells and dendritic cells in the skin [68]. These molecular results of IL-17A inhibition through secukinumab were further elucidated in 2017 by Kolbinger and colleagues [69], who looked at protein levels and gene expression in the skin of eight patients treated with secukinumab. Among the top 10 downregulated proteins after 8 and 15 days were IL-1β, AMPs (β-defensin 2 and LCN2), neutrophil enzyme MPO, neutrophil chemoattractants (CXCL-1 and CXCL-5), and Th17 chemoattractant CCL-20. Early downregulation of gene expression was observed in AMPs (*BD2*, *LCN2*, *LL37*, *S100A8*, and *S100A9*) as well as neutrophil- and Th17-attracting chemokines (*CXCL1*, *CXCL8*, and *CCL20*) and the IL-1 family member *IL36A*. Serum protein levels of IL-17A and β-defensin 2 correlated with PASI reduction in the treated patients, suggesting that serum β-defensin 2 in particular could be a potential biomarker for response to secukinumab [69].

Brodalumab is an IL-17RA-targeting monoclonal antibody that has shown high clinical efficacy [30].Transcriptional analysis of psoriatic skin samples conducted by Russel et al. [70] revealed that brodalumab, similar to other biologics, induces early changes in keratinocyte markers after IL-17RA inhibition, accompanied by a delayed response of T cell and leukocyte genes. Within 2 weeks after start of treatment, keratinocyte genes such as *IL36A*, *S100A7*, *KRT6*, *CXCL6*, and *IL17C* had decreased markedly, and certain inflammatory cytokines such as *IL12A* and *IL23A* were normalized, while the expression of *IL17A*, *IL17F*, and *IL22* was only partially reduced and decreased more substantially after 6 weeks. Also, as in other studies with biologics, epidermal proliferation was reduced quickly, followed by a slow decrease in T cell counts in the skin and complete normalization at 6 weeks after start of brodalumab treatment. In addition, the investigators generated and compared gene expression scores for keratinocyte genes induced by either IL-17 or IFNγ during the course of brodalumab treatment. While the IL-17 score almost returned to levels seen in non-lesional skin, the IFNγ score only partially diminished. Clinical improvement of psoriasis correlated with normalization in the expression of IL-17-induced keratinocyte genes. Thus, IL-17R blockade by brodalumab may directly target keratinocytes, since there is a fast normalization of keratinocyte markers and slower changes in cytokines produced by T cells, as well as T cell numbers in the skin after treatment. The slow decrease of IL-22 and remaining high levels of IFNγ imply that clinical and molecular improvement through brodalumab treatment depends little on changes in Th1 and Th22 cells [70].

### Anti-IL-23 treatment

The cytokines IL-12 and IL-23 are both heterodimers, sharing the IL-12/IL-23p40 subunit, and additionally consist of IL-12p35 and IL-23p19, respectively. Both cytokines are produced by myeloid dendritic cells and influence T cell differentiation [71]. Since IL-23 helps to drive psoriasis, several biologics have been designed that target its subunits, p40 (targeted by ustekinumab) and p19 (targeted by guselkumab, risankizumab, and tildrakizumab). Brodmerkel et al. [72] compared global mRNA expression changes by microarray analysis in psoriatic skin samples from patients treated and achieving PASI75 with the anti-IL-12/IL-23p40 drug ustekinumab versus with etanercept. Within 12 weeks, both ustekinumab and etanercept caused a significant change in 5000 genes and 4500 genes, respectively. While the majority of differentially expressed genes (DEGs) were shared by both drugs, inhibition of IL-12/IL-23p40 uniquely modulated 700 genes and suppression of TNFα uniquely altered expression of around 400 other transcripts. Looking more closely at prominent psoriasis genes, a strong downregulation of IL-17-related genes (*IL19*, *DEFB4*, *CCL20*, *LCN2*, *IL1B*) and a less impressive but still significant reduction of IL-17A and IL-23 subunit genes were observed after treatment with both drugs. However, overall psoriasis-associated genes and genes belonging to signaling pathways of IL-22, IFNγ, TNFα, IL-1, and IL-17 were more strongly suppressed by ustekinumab than by etanercept. While a stronger effect on IFNγ-associated genes by IL-12/IL-23p40 inhibition was anticipated, the strong impact on TNF-associated genes was surprising.

The term “molecular scar” has been used for residual disease genes that are less than 75% suppressed from baseline levels by treatment [62]. After ustekinumab treatment, the expression levels of 18% of disease-related genes did not return to baseline levels compared with 23% after etanercept at similar clinical efficacy. This led Brodmerkel et al. to conclude that the smaller molecular scar left after ustekinumab treatment may create a more stable environment for resolved lesions, resulting in a lower likelihood of disease recurrence due to reduced IL-17 expression by T cells induced by blockade of IL-23. Etanercept, on the other hand, blocks IL-23 only indirectly, as it targets IL-23-producing dendritic cells by diminishing the action of TNFα [72] and thus may have a weaker effect on IL-17 levels.

Visvanathan and colleagues compared the molecular scar left after treatment with the IL-23p19 inhibitor risankizumab versus with ustekinumab [73]. Clinically, risankizumab is overall more effective than ustekinumab [27, 73]. To determine why, cellular markers and gene expression profiles were compared in skin samples from patients treated with the drugs. Both drugs lessened epidermal thickness and reduced protein levels of K16, CD3, CD11c, DC-LAMP, Ki67, S100A7, LCN2, and β-defensin-2 after 4 weeks. Transcriptional analyses showed that both biologics led to early decreases of IL-17/IL-23 pathway genes (e.g., *IL17A*, *IL17F*, *IL17C*, *IL22*, *IL23A*, *S100A8*, *S100A9*, *LCN2*, and *BD2*). Risankizumab, however, had a stronger decreasing effect on genes that were upregulated in reconstructed epidermal cells and keratinocytes after in vitro stimulation with IL-17 [73]. Differences were also observed in psoriasis-associated genes related to keratinocytes, monocytes, and macrophages. Overall, risankizumab had a stronger effect on disease-related genes than ustekinumab did, although PASI reduction after 4 weeks was similar [73].

Guselkumab is another antibody against IL-23p19 approved for treatment of psoriasis. In 2014, Sofen et al. [74] analyzed effects of IL-23p19 inhibition in 24 psoriasis patients after 1 and 12 weeks of treatment. After 1 week, only slight changes in epidermal thickness and CD3 and CD11c positive cells were found. After 12 weeks, epidermal thickness as well as protein expression of keratin 16 and CD3 and CD11c cell counts in the skin were significantly reduced. A significant reduction of epidermal hyperplasia gene *KRT16* and IL-17 pathway-related genes *S100A7*, *LCN2*, *CXCL1*, and *CXCL8* and a modest reduction of *IL17A* were established by qPCR. Genome-wide transcriptional analysis revealed normalization by 70% or more of almost all disease-related genes (about 1200 genes) after 12 weeks of guselkumab treatment. In fact, the mRNA profile after 12 weeks of IL-23p19 inhibition closely resembled that of non-lesional skin. To further extend their findings, Sofen et al. performed serum immunoassays. Interestingly enough, protein levels of IL-17A were already strongly reduced after 1 week and then further decreased at 12 weeks. However, guselkumab did not affect serum levels of various other psoriasis-related cytokines, including IL-23p19 [74].

In sum, blocking solely the p19 subunit of IL-23 and not both p19 and p40 is a more effective approach in the treatment of psoriasis. Aside from neutralizing IL-23, which is thought to be responsible for the curative effect, anti-p40 therapy also interferes with IL-12 signaling and type 1 immunity [75]. Using a preclinical model for psoriatic plaque formation Kulig et al. [75] showed that IL-12, in contrast to IL-23, had a regulatory function by restraining the invasion of an IL-17-committed γδT (γδT17) cell subset and that IL-12 receptor signaling in keratinocytes initiated a protective transcriptional program that limited skin inflammation. Thus, therapeutic collateral targeting of IL-12 and IL-23 may be counterproductive in the therapy of psoriasis. Indeed, the findings by Kulig et al. help to explain the therapeutic inferiority of IL-12/23 inhibition by ustekinumab to pure anti-IL-23 inhibition by risankizumab, guselkumab, or tildrakizumab.

## Cellular scar

The fact that psoriasis recurs most often at the very same body sites where the initial lesion occurred suggests that critical cells may be left behind in the skin in situ after treatment is stopped. In this regard, Cheuk and coworkers [76] reported the results of an elegant study in 2014, addressing the question whether psoriatic lesions harbor tissue-resident memory T cells (TRMs) that could possibly drive disease recurrence at the same, i.e., initial site of lesions. They sampled narrow band-UVB-treated, anti-TNFα or anti-IL-12/23-treated healed psoriatic skin and studied gene expression and ex vivo cytokine production by T cells. Interestingly, they found that approximately half of CD8+ T cells in the epidermis of active lesions expressed TRM markers CD103 and CD49a. This suggested an expansion of TRMs at diseased body sites of psoriasis compared with healthy skin. In resolved lesions, epidermal CD4+ T cells had returned to normal levels, while numbers of dermal CD4+ and CD8+ T cells were still elevated. Although epidermal CD8+ T cells were decreased, the number of CD49a-expressing CD8+ T cells was still higher than in non-lesional or healthy skin (that had not had visible psoriatic manifestation). Transcriptional analysis of isolated T cells from resolved psoriatic lesions showed that epidermal T cells had higher expression of Th17-associated genes (*RORC*, *IL17A*, *IL22*) than did dermal T cells. Upon ex vivo stimulation, dermal T cells from those lesions had a cytokine production profile similar to that found in healthy skin. Interestingly, the percentage of IL-22+ epidermal CD4+ T cells from resolved lesions was similar to that in active lesions. Epidermal CD8+ T cells from resolved lesions produced more IL-17A after ex vivo stimulation than did those from healthy skin, indicating that these cells are still functional even after long-term anti-psoriatic treatment. Epidermal IL-17-producing CD8+ T cells from healthy skin, lesional psoriasis, and resolved psoriasis expressed CD103, suggesting that these are in fact TRMs. In resolved lesions, an epidermal subpopulation of CD8+ T cells co-expressed TRM marker CD103 and Th17 markers IL-23R and CCR6, indicating responsiveness to IL-23 signaling. Moreover, Cheuk et al. [77] found two subsets of CD8+ TRMs in the epidermis, IL-17-producing CD49- and IFNγ-producing CD49+ TRMs. Intriguingly, IL-17-producing CD49− TRMs from the epidermis showed higher IL-17 production than did CD49− TRMs isolated from dermis. This suggested that distinct functional subsets exist in epidermis and in dermis. In active psoriasis, some CD49− TRMs as well as some CD49+ TRMs expressed both IL-17 and IFNγ. However, after isolation and ex vivo stimulation, these cells did not produce IL-17. Thus, the investigators speculated that T cell receptor (TCR) activation might be needed in addition to inflammatory signals promoting IL-17 response [77].

Most recently, Matos and co-workers [78] used immunofluorescence staining and high-throughput sequencing (HTS) of the CDR3 domain of the T cell receptor as a different approach to assess density and clonality of T cells in order to study residual T cells in resolved psoriatic lesions. TCR sequencing indicated that there were more T cells in resolved psoriatic lesions than in the skin of healthy controls and non-lesional skin of psoriasis patients; in fact, levels in resolved lesions were similar to those in active lesions. While diverse T cell clones were present in active lesions before treatment, only 7% of T cell clones remained detectable in clinically healed lesions. This suggests that, when inflammation subsides, the majority of T cell clones leave the skin. Increased numbers of oligoclonal T cell populations were found not only in resolved lesions but also in non-lesional skin. When T cells were isolated from resolved lesions to study their cytokine production ex vivo, expanded T cell clones from both active and clinically healed lesions produced IL-17A. TCR sequencing also showed that putative pathogenic T cell clones were αβT cells (implying that their TCR is composed of α- and β-chains) and not γδT cells. Furthermore, unique αβ TCR sequences that were identified in the skin of psoriasis patients were absent from the skin of healthy controls and patients with other skin diseases. These findings implied that, in psoriasis, increased T cell response might be due to the presence of a common antigen. Pathogenic T cell clones producing IL-17A and residing in low numbers in clinically healed lesions could in fact be TRMs. They may be resistant to elimination by therapy and bear the potential to restart the psoriatic inflammatory feedback loop at the site of residence [78].

In 2018, Gallais-Sérézal and colleagues studied how resolved psoriatic tissue responded to activation of putative pathogenic TRMs. Using sampled skin biopsies from healthy controls and resolved lesions from psoriasis patients, they produced skin explant cultures and then stimulated them with anti-CD3 antibody OKT-3 to activate resident T cells. The skin environment after T cell activation was analyzed by NanoString transcriptional methodology, and IFNγ-related signaling pathways were found to be upregulated in both healthy and resolved psoriatic skin. However, in resolved psoriasis alone (and not in healthy skin), IL-17 signaling was upregulated in the epidermal compartment. This implied that renewed activation of resident T cells in resolved lesions leads to keratinocyte activation, which could translate into chemokine release and recruitment of circulating leukocytes. Indeed, IL-17 signaling-related tissue-response correlated with clinical relapse after therapy, thus highlighting the key role of pathogenic resident T cells in psoriasis [79]. The same group of investigators studied TRMs in non-lesional skin from psoriasis patients to determine whether these resident T cells are indeed pathogenic and have the ability to induce psoriasis in never-lesional skin. Indeed, ex vivo T cell activation of skin explants led to psoriasis-typical tissue responses as determined by NanoString analysis. Additionally, CD103+ CD8+ T cells and CCR6+ CD4+ T cells were enriched in non-lesional skin of psoriasis patients compared with that in healthy controls. Moreover, CCR6+ CD4+ T cells were capable of producing IFNγ and IL-17. These results suggested that pathogenic TRMs do exist in non-lesional skin [80].

Although pathogenic TRMs have certainly gained more attention in recent years, some progress has been made in analyzing the presence and function of DCs and Langerhans cells in resolved psoriasis as well. While DC numbers were found to be high in active psoriatic lesions and absent from resolved skin, the data on Langerhans cells are controversial [81, 82]. Langerhans cells from active lesions expressed psoriasis-associated genes (*IL23*, *IL1B*, *IL15*) and showed increased IL-23 production after ex vivo stimulation. In resolved lesions, expression of *IL23* and *IL15* was still higher than in healthy skin. Upon ex vivo stimulation, these cells were still capable of producing IL-23 [83]. In a comprehensive review on the role of Langerhans cells in psoriasis, Eidsmo and Martini [81] hypothesized that Langerhans cells are not only found in close proximity to T cells, but are also able to cross-talk with both T cells and keratinocytes. This, together with the ability of Langerhans cells to produce IL-23, implies that Langerhans cells could play a role in renewed activation of T cells and disease recurrence.

Last but not least, Johnson-Huang and colleagues [46] showed that NB-UVB leads to clearance of psoriasis by suppressing the IL-17/IL-23 axis. They compared responders vs. non-responders after 6 weeks of NB-UVB therapy and looked at myeloid DCs and T cells and their inflammatory products using immunohistochemistry and qPCR. Inflammatory myeloid DC levels were reduced and expression of DC-related cytokines (e.g., *INOS*, *IL20*, *IL23p19*, *IL12/IL23p40*) was decreased in responsive plaques. Furthermore, CD3 T cell counts were reduced in responders, and cell counts strongly correlated with the defined “response score”. In addition, expression of *IFNG*, *IL17*, *IL22*, and IL-17/IL-22 downstream genes β-defensin 4 (*BD4*) and myxovirus-resistance 1 (*MX1*) was significantly suppressed. All of these changes were not observed in non-responsive plaques. Interestingly, the “response score” correlated with *IL17* and *IL22* expression, but not with *IFNG* expression. To determine whether these results show a direct effect of NB-UVB or merely changes that occur when psoriasis clears up in general, Johnson-Huang’s group performed an in vitro experiment where PBMCs were isolated, treated with NB-UVB, and measured for cytokine expression after 4 h. Intracellular staining showed that production of IFNγ was suppressed by 85% and that IL-17 and IL-22 produced by CD3+ T cells were decreased by 45% and 89%, respectively [46]. It is fascinating to learn that a nature-derived therapy such as UVB phototherapy seems to interfere with the very same pathway targeted by today’s most modern drug treatments, i.e., the IL-17/23 axis.

## Conclusion and open questions

**Fig. 3:**
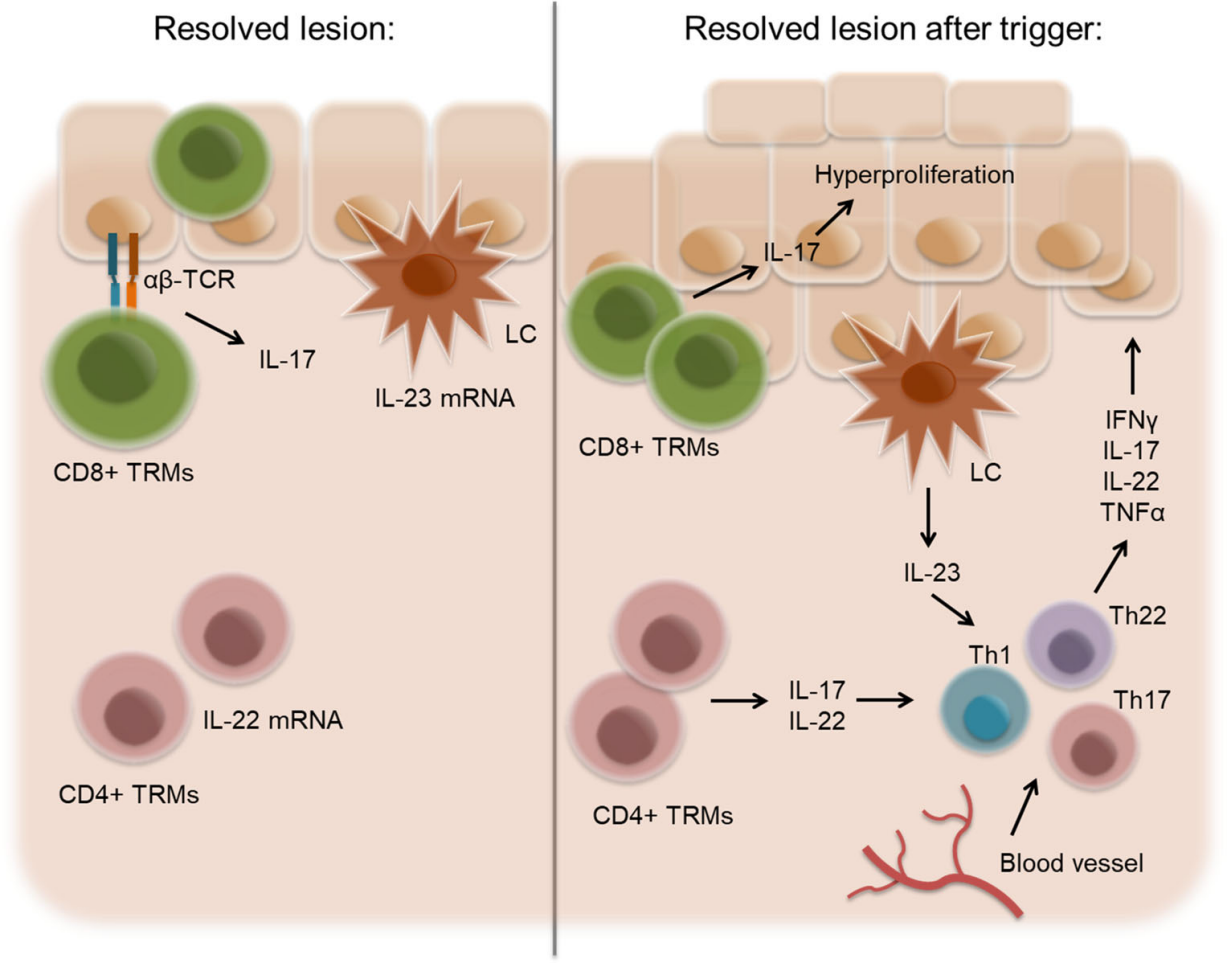
Model mechanism for disease recurrence in resolved lesions. In clinically healed lesions, CD4+ T cells remain in the dermis and express IL-22 mRNA. Langerhans cells (LCs) residing in the epidermis express IL-23 mRNA. Epidermal CD8+ TRMs expressing αβTCR are able to produce IL-17. Upon disease trigger, LCs and T cells actively produce pro-inflammatory cytokines and cause recurrent inflammation

Numerous studies have shown that the molecular imprints of psoriasis do not fully resolve in macroscopically cleared skin even after the most efficacious targeted treatment available nowadays, including anti-IL-17 and anti-IL-23 antibody administration. Resolved lesions have a molecular scar composed of about 250 gene products that are not fully normalized after treatment. These transcripts can be grouped into inflammation-associated genes such as *IL17*, *IL22* and others and skin structure-related genes [62]. Moreover, pathogenic memory T cells seem to persist at sites of clinically resolved psoriatic lesions. Even after long-term therapy, these cells do not lose their ability to produce IL-17A [76, 78], which can signal to keratinocytes and stimulate their proliferation [80]. Although the role of innate cells has not been studied extensively, Langerhans cells isolated from resolved psoriatic lesions can produce IL-23 after stimulation, which makes them another potential player in the restart of activation of “sleeping” TRMs [83] and possible reappearance of psoriatic lesions [76] (Fig. 3). The question now is this: what initially triggers (or retriggers) the process of psoriatic recurrence after a treatment has been stopped? Could IL-17 be stored and hidden in certain cells of the skin, perhaps mast cells, waiting to be released after endogenous and/or exogenous stimulation to activate the inflammatory loop again? What about the neurogenic inflammation of the skin [84] that plays an essential role at least in the itch of psoriasis? Might a nerve scar left behind after successful treatment contribute to the reappearance of psoriatic lesions, be it during or after continuous treatment? Last but not the least, may the interplay of the microbiome and AMPs [85, 86] help trigger psoriatic recurrence? This may be in particular important for pustular psoriasis, in which IL-1/IL-36 plays an important role in the pathogenesis [87–89]. A vicious loop between AMPs such as cathelicidin (LL-37) and IL-36 signaling may drive psoriatic disease [90–93]. In fact, IL-36 receptor blockade revealed promising results in pustular psoriasis [94] and may also be a therapeutic option in plaque-type psoriasis [95]. Future work will have to address all of these open questions in order to improve and advance overall treatment strategies for psoriasis and allow long-term, continuous disease control.
